# Sensation Seeking’s Differential Role in Face-to-Face and Cyberbullying: Taking Perceived Contextual Properties Into Account

**DOI:** 10.3389/fpsyg.2019.01572

**Published:** 2019-07-16

**Authors:** Daniel Graf, Takuya Yanagida, Christiane Spiel

**Affiliations:** Department of Applied Psychology, Work, Education and Economy, Faculty of Psychology, University of Vienna, Vienna, Austria

**Keywords:** bullying, cyberbullying, sensation seeking, need for stimulation, avoidance of rest

## Abstract

Several studies have demonstrated a relationship between sensation seeking and aggression. However, few studies have examined the relationships between sensation seeking and face-to-face and cyberbullying. The few existing studies assessed sensation seeking with items partly referring to antisocial behavior. This could have led to tautological findings. Moreover, contextual properties that could account for differences between bullying contexts (face-to-face, cyberspace) were neglected. Therefore, the first goal of this study was to investigate the relationships between sensation seeking and face-to-face and cyberbullying in a way that avoids tautological findings. Thus, sensation seeking was operationalized as a motivational disposition encompassing the dimensions “need for stimulation” and “avoidance of rest.” Furthermore, students’ perceptions of the contextual properties of the face-to-face and cyber context and their relevance for the relationships between the dimensions of sensation seeking and face-to-face and cyberbullying were examined. A total of 523 students (*M*_age_ = 17.83; *SD* = 2.13; ♀ = 37.4%) from four vocational schools answered online questionnaires on face-to-face and cyberbullying involvement, perceived contextual properties, and the two dimensions of sensation seeking during regular school hours. Structural equation modeling revealed positive associations between need for stimulation and both forms of bullying. Avoidance of rest, however, was positively related to cyberbullying only. The differences in all regression slopes between contexts were statistically significant. That is, the positive associations with the two dimensions of sensation seeking were stronger for cyberbullying than for face-to-face bullying. Dependent *t*-tests revealed differences in students’ perceptions of contextual properties between contexts (face-to-face, cyberspace). Nevertheless, no significant relationships between either dimension of sensation seeking and either form of bullying were moderated by any perceived contextual property. Our results demonstrate sensation seeking’s greater role in cyberbullying and confirm differences in perceived contextual properties between the face-to-face and cyber context. Furthermore, the fact that no perceived contextual property moderated the significant relationships between the dimensions of sensation seeking and face-to-face or cyberbullying shows the relatively greater role of a single person factor compared to single contextual properties.

## Introduction

Research on human aggression investigates both contextual and person factors to identify the causes and conditions for the emergence of aggressive behavior (Anderson and Bushman, 2002). Among person factors, sensation seeking is a frequently studied risk factor for engaging in aggressive behavior, and research repeatedly has shown positive relationships between sensation seeking and aggression (Zuckerman, 2007; Wilson and Scarpa, 2011; Bacon et al., 2014). However, although bullying is seen as a subset of aggression (e.g., Smith, 2004), little is known about the role of sensation seeking in bullying (Kowalski et al., 2014).

Bullying is considered a complex social phenomenon (Simon and Nail, 2013) and is defined as aggressive behavior that is intended to hurt another individual (Berkowitz, 1993). In addition to aggressive behavior, bullying involves a power imbalance and repetitiveness (Olweus, 1993). Bullying behavior can take various forms, such as physical, verbal, or relational (Olweus, 2013). Moreover, in light of the spread of new information and communication technologies (ICTs) and the differences in contextual properties between face-to-face and computer-mediated communication (CMC; see, for example, Gunawardena, 2004), there is growing evidence that considering the context (face-to-face, cyberspace) in which bullying occurs is of great importance (Suler, 2004; Runions, 2013; Runions and Bak, 2015; Graf et al., 2019). As it is unclear whether bullying in the face-to-face context and bullying via ICTs (i.e., cyberbullying) can be considered equivalent (Olweus, 2012), investigating contextual differences between face-to-face and cyberbullying regarding the role of risk factors (i.e., sensation seeking) is crucial in order to inform the development of evidence-based prevention and intervention strategies.

### Sensation Seeking

Sensation seeking is “… defined by the seeking of varied, novel, complex, and intensive sensations and experiences, and the willingness to take physical, social, legal and financial risks for the sake of such experiences” (Zuckerman, 1994, p. 27). Sensation seeking can be explained by genetic, biological, psychophysiological, and social factors (Zuckerman, 1994, 1996), and sensation seekers are described as individuals who engage in behaviors to increase the amount of experienced stimulation, thus seeking out arousal (Roberti, 2004). According to sensation seeking theory (Zuckerman, 1979), this might be due to a chronic low arousal state that is perceived as aversive. Individuals who suffer from this low state of arousal seek out stimulating situations in order to increase their arousal level to their personal optimum. In this context, some authors argue that sensation seeking comprises both socialized and unsocialized modes, with the latter leading to aggressive behavior to a certain extent (Glicksohn and Abulafia, 1998). Consequently, low levels of arousal have been found in face-to-face bullies (Woods and White, 2005).

However, Arnett (1994) emphasizes that environmental factors may shape the expression of sensation seeking. For example, Rogers et al. (2018) found person–context interactions in sensation seeking-related alcohol use. These authors demonstrated that the relationship between sensation seeking and alcohol use was more pronounced for adolescents who lived in less structured environments. Moreover, based on a review of behavioral and biological correlates of sensation seeking, Roberti (2004) argues that sensation seekers prefer contexts in which they can participate in activities suitable to their needs. Thus, given the contextual differences between face-to-face communication and CMC, differential relationships between sensation seeking and face-to-face and cyberbullying might also be conceivable.

In any event, a person may gratify their tendency to seek out stimulating experiences in different areas, such as in occupational, recreational, sports, and social interactions (Roberti, 2004). Consequently, sensation seeking is related not only to aggressive behaviors, but also to a variety of other risky behaviors, such as substance use and risky driving (Crawford et al., 2003; Dunlop and Romer, 2010). However, while sensation seekers may tend to find themselves in risky situations, risk is a correlate and not the primary motive of sensation seeking (Zuckerman, 1994). Instead, sensation seeking is thought to be an appetitive and primarily reward-related motivational construct (Steinberg, 2008; Steinberg et al., 2008; Runions, 2013). Thus, for high sensation seekers, rewarding goal states are states of stimulation, whereas situations characterized by rest might be perceived as unpleasant (Roth and Hammelstein, 2012). In accordance with these considerations, Roth and Hammelstein (2012) postulated two dimensions of sensation seeking as a motivational disposition: “need for stimulation” and “avoidance of rest.”

### Bullying

Whereas aggressive behavior is defined as behavior with the intention to harm another person (Bushman and Anderson, 2001), bullying – as a prevalent subtype of aggressive behavior – must additionally happen repeatedly in a situation characterized by a power imbalance (Olweus, 1993; Smith and Ananiadou, 2003). Moreover, with the spread of ICTs in recent years, new bullying practices have emerged that occur online (e.g., editing and publishing embarrassing pictures and videos). Research on bullying via ICTs, or cyberbullying, has largely adopted the paradigms developed in face-to-face bullying research and typically defines this form of bullying using the same criteria such as in face-to-face bullying (Smith et al., 2008; Tokunaga, 2010; Vivolo-Kantor et al., 2014).

Just as with sensation seeking, rewards are thought to play a significant role in (cyber)bullying: Both forms of bullying are often referred to as an instrumental, proactive, and deliberate act of aggression that is used to gain resources (e.g., Crick and Dodge, 1996; Sutton et al., 1999; Roland and Idsøe, 2001; Gradinger et al., 2012). Consequently, face-to-face and cyberbullying behavior is mostly seen as planned, unprovoked, and goal-directed behavior related to the anticipation of rewarding outcomes such as social dominance, non-social resources (e.g., wealth), or reproductive gains (Volk et al., 2014). Immediate affective rewards, such as excitement and thrill, have also been discussed as motives for engaging in face-to-face and cyberbullying behavior (Howard, 2011; Runions, 2013).

### Differences Between Face-to-Face and Cyberbullying

Applying the same framework to bullying in the face-to-face and cyber contexts may lead the influences of context-inherent properties on (cyber)bullying behavior to be overlooked (Suler, 2004; Vandebosch and Van Cleemput, 2008; Dooley et al., 2009; Menesini and Nocentini, 2009; Runions, 2013; Runions and Bak, 2015). From the perpetrator’s perspective, cyberbullying might be seen as a more convenient form of bullying due to properties of ICTs (Antoniadou and Kokkinos, 2015). For example, CMC makes it possible to act anonymously, reducing one’s accountability (Kowalski and Limber, 2007; Dooley et al., 2009). Moreover, authorities such as parents and teachers might have less of a presence in the cyber context compared to the face-to-face context and might therefore underestimate their own children’s involvement in cyberbullying incidents (Dehue et al., 2008). In terms of social rewards, the perceived audience size may be seen as potentially bigger in cyberspace than in the face-to-face context (Slonje et al., 2013; Kowalski et al., 2014), which can function as an incentive to act out during adolescence (Steinberg, 2005; Chein et al., 2011). Furthermore, others’ reactions are delayed in CMC compared to face-to-face communication (Sourander et al., 2010; Kowalski et al., 2012a,b). In some cases, there is even a complete lack of reactions by others in cyberspace. From the perpetrator’s perspective, this lack of reaction may prevent empathy from being triggered, inhibiting feelings of remorse (Slonje et al., 2012) and thus facilitating continued cyberbullying (Graf et al., 2019). In this study, we addressed adolescents’ actual perceptions of these contextual properties (i.e., perceived anonymity, lack of authorities, audience size, and immediacy of reactions by others) when communicating in cyberspace and in face-to-face context, thus complementing previous largely theoretically-derived conceptual analyses (e.g., Kowalski et al., 2012b, 2014; Runions, 2013; Runions et al., 2013).

### Sensation Seeking and Face-to-Face and Cyberbullying

Only a few studies have examined the relationships between sensation seeking and face-to-face and cyberbullying simultaneously (e.g., Antoniadou et al., 2016). Their findings suggest that sensation seeking is a common correlate of bullying in both contexts (e.g., Antoniadou et al., 2016). However, the aim of these studies was to identify common predictors for face-to-face and cyberbullying. They did not focus on the relationships between (cyber)bullying and sensation seeking *per se* but investigated them alongside other assumed predictors. These studies measured sensation seeking with Zuckerman’s Sensation Seeking Scale Form V (SSS-V; Zuckerman et al., 1978) or adapted forms (e.g., SSS-A; Hoyle et al., 2002), even though the assessment of sensation seeking with the SSS-V has been criticized repeatedly (e.g., Arnett, 1994; Roth et al., 2007) due to the inclusion of items describing concrete antisocial behavior. According to Roth et al. (2007), this may lead to tautological findings due to the conflation of predictors (e.g., sensation seeking) and outcomes (e.g., bullying). To avoid tautological findings, the authors suggest operationalizing sensation seeking as a motivational disposition focusing on the aim of a behavior and not on the behavior *per se*.

However, although differential relationships between sensation seeking and face-to-face and cyberbullying are conceivable, to the best of our knowledge, no study has investigated the different dimensions of sensation seeking as a motivational disposition in relation to face-to-face and cyberbullying so far. For example, bullying behavior is seen as more convenient in cyberspace than in the face-to-face context (e.g., fewer authorities, more anonymity, a larger perceived audience may lead to higher anticipated rewards, a lack of or delayed reactions by others hamper or inhibit empathy and remorse), which may facilitate sensation seeking-related cyberbullying. Additionally, in cyberspace, the set of potential actions that sensation seekers can take to increase their arousal to a personal optimal level may be restricted. Thus, sensation seekers may engage in unsocialized modes of sensation seeking more often in cyberspace than in the face-to-face context, again facilitating sensation seeking-related cyberbullying.

### The Present Study

Although it has been theoretically discussed in the literature (e.g., Runions, 2013), there is still a lack of empirical evidence on how sensation seeking may be differentially related to engagement in face-to-face and cyberbullying. Therefore, the aim of our study was to examine whether sensation seeking relates to face-to-face and cyberbullying in similar or different ways. To ensure that we did not measure concrete antisocial behaviors within the construct of sensation seeking, we operationalized sensation seeking along two dimensions, in accordance with Roth and Hammelstein (2012): “need for stimulation” and “avoidance of rest.”

Moreover, taking into account the proposed role of environmental factors for the expression of sensation seeking-related behavior and aiming to gain deeper insight into contextual differences between face-to-face and cyberbullying, we were further interested in whether perceived contextual properties (i.e., anonymity, audience size, lack of authorities, and immediacy of reactions by others) were perceived differently in face-to-face communication versus CMC, and whether these perceived contextual properties moderated the associations between the two dimensions of sensation seeking and face-to-face and cyberbullying.

First, differential relationships and differences in the strength of these relationships between the dimensions of sensation seeking and face-to-face and cyberbullying would indicate that sensation seeking is differentially relevant for bullying in the two contexts (face-to-face, cyberspace). Second, differences in perceived properties between contexts (face-to-face, cyberspace) may shed light on the contextual properties that are relevant for these differential relationships. Third, investigating interactions between the dimensions of sensation seeking and the perceived contextual properties of face-to-face and cyberbullying may contribute to our understanding of how perceived contextual properties may affect these relationships.

On the basis of previous research (e.g., Wilson and Scarpa, 2011; Antoniadou et al., 2016), we hypothesized positive relationships between the two dimensions of sensation seeking and face-to-face and cyberbullying (Hypothesis 1). We further hypothesized a stronger association between the two dimensions of sensation seeking and cyberbullying compared to face-to-face bullying (Hypothesis 2).

Moreover, we assumed that anonymity is perceived as higher in the cyber than in the face-to-face context (Hypothesis 3a). We further hypothesized that authorities are perceived as more present in the face-to-face than in the cyber context (Hypothesis 3b), that audience size is perceived as larger in the cyber than in the face-to-face context (Hypothesis 3c), and finally, that reactions by others are perceived as more immediate in the face-to-face than in the cyber context (Hypothesis 3d).

Next, we hypothesized positive moderating effects of perceived anonymity, perceived audience size, and perceived lack of authorities on the relationships between both dimensions of sensation seeking and face-to-face and cyberbullying (Hypotheses 4a–4c). Finally, we assumed that the higher the perceived immediacy of reactions by others, the weaker the relationship between the dimensions of sensation seeking and both forms of bullying (Hypothesis 4d).

## Materials And Methods

### Procedure and Sample

This study was embedded in a larger survey on intrapersonal risk and protective factors for face-to-face and cyberbullying. Distinct research questions (i.e., the role of empathy in face-to-face and cyberbullying) have been examined and published before (see Graf et al., 2019). We randomly invited the school principals of 39 Lower Austrian vocational schools to participate in our study. Four of them agreed. We chose to conduct our study in vocational schools, as evidence suggests higher self-reported bullying rates for vocational school students than students enrolled in traditional schools (e.g., Menesini et al., 2009; Zych et al., 2017). This study was approved and supported by the school board of the federal state of Lower Austria. The federal state school board and the participating school principals ensured that parental consent was given in accordance with the school board’s official guidelines. According to these guidelines, parents’ informed consent in written form is not required for vocational school students.

A total of 523 students (37.4% girls; *M*_age_ = 17.83 years; *SD* = 2.13; age range 15–28 years) from 32 school classes answered online questionnaires during regular school hours in their school’s computer lab. Research assistants were present at all times. Participation was voluntary, participants gave informed consent, and the consent rate was above 99%. The age distribution of the sample is shown in Table 1.

**TABLE 1:** Age distribution of the sample.

**Age in years**	**15**	**16**	**17**	**18**	**19**	**20**	**21**	**22**	**23**	**24**	**25**	**26**	**27**	**28**
Frequencies	38	111	115	111	59	35	15	14	10	4	1	3	2	1
Percentages (%)	7.3	21.4	22.2	21.4	11.4	6.7	2.9	2.7	1.9	0.8	0.2	0.6	0.4	0.2

*Percentages have been rounded and may not total 100%.*

### Measures

Below, we present the measures we used in our survey.

#### Sensation Seeking

We assessed self-reported sensation seeking with the Need Inventory of Sensation Seeking (NISS, Roth and Hammelstein, 2012). Following need theory (Cattell, 1979), the NISS focuses on a psychological or physical sensation as a goal state rather than assessing concrete behaviors. The NISS comprises 17 items measuring two dimensions, namely need for stimulation (11 items, e.g., “I like the feeling of excitement in my body”) and avoidance of rest (six items, e.g., “I like to just sit back and enjoy a peaceful moment”). Participants had to indicate on a five-point response scale (1 = almost never, 2 = rarely, 3 = sometimes, 4 = often, 5 = almost always) how often they felt the way described in the given statements. In this study, Cronbach’s alpha was α = 0.86 for need for stimulation and 0.78 for avoidance of rest.

#### Cyberbullying and Face-to-Face Bullying

Self-reported cyberbullying and face-to-face bullying behavior was assessed with the European Cyberbullying Intervention Project Questionnaire (ECIPQ; Del Rey et al., 2015). On a five-point response scale (1 = no, 2 = yes, one or two times, 3 = yes, one or two times per month, 4 = yes, approximately one time per week, 5 = yes, more than once a week), students had to indicate whether they had intentionally engaged in cyberbullying (example item: “I hacked into someone’s account and stole personal information”) or face-to-face bullying (example item: “I hit, kicked, or pushed someone”) behavior within the last 2 months. The cyberbullying scale includes 11 items and the face-to-face bullying scale 7 items. The ECIPQ has been structurally validated in six countries (Del Rey et al., 2015). In this study, Cronbach’s alpha was α = 0.95 for cyberbullying and 0.89 for face-to-face bullying.

#### Perceived Contextual Properties

We measured perceived contextual properties of face-to-face communication and CMC using semantic differentials. The semantic differential is a measurement technique allowing for the measurement of evaluative judgments by presenting bipolar attributes (Osgood et al., 1957). This procedure is frequently used in environmental research (e.g., Humpel et al., 2004; Michon et al., 2005; Fielding et al., 2008). We decided to use semantic differentials because we assumed high face validity with respect to the measurement of the intended contextual properties and sought to avoid socially desirable answers and acquiescence bias (see, for example, Friborg et al., 2006). We generated the statements used in this study on the basis of the considerations outlined in the section “Differences Between Face-to-Face and Cyberbullying.” On a five-point response scale, students had to indicate their perceptions of the context when communicating on the Internet/with smartphones or face-to-face (not on the Internet or with smartphones). Two opposite statements were presented for each property, and participants had to choose where their own position lay between them. The statements for perceived anonymity were “You will be recognized quickly” and “You will remain unrecognized.” The statements presented to measure perceived audience size were “You have a small audience” and “You have a large audience.” The statements measuring perceived lack of authorities were “There are many people who can punish you” and “There are few people who can punish you.” Perceived immediacy of reactions by others was assessed by presenting the statements “You notice others’ reactions to your own behavior very slowly” and “You quickly notice others’ reactions to your own behavior.”

#### Covariates

We included gender and age as covariates, as research has shown higher prevalence rates of face-to-face bullying among boys and younger adolescents (Kowalski et al., 2014). Moreover, as there is evidence that social media use effects cyberbullying (Best et al., 2014), we considered social media use by asking participants how often they check social media right after waking up on a five-point response scale (1 = never, 2 = rarely, 3 = sometimes, 4 = often, 5 = always).

### Missing Data

A total of 0.12% of data were missing, stemming from 21 incomplete records. The percentage of missing values across the 46 variables ranged from 0.00 to 2.87%.

A series of two-sample Wilcoxon tests with continuity correction and Bonferroni–Holm correction for multiple comparisons were conducted as a missing data analysis. The results revealed no differences between students with complete and incomplete data on any variable (effect sizes ranged between *r* = 0.00 and *r* = 0.16).

Full information maximum likelihood (FIML) under the missing at random (MAR) assumption was used to deal with missing data (see Enders, 2010).

### Measurement Models

Confirmatory factor analysis (CFA, see Brown, 2015) was conducted in Mplus 8.1 (Muthén and Muthén, 1998–2018) to test the measurement models for the present study. CFA with ordered categorical indicators using robust weighted least squares estimator (WLSMV) was applied in order to take into account the ordered categorical nature of the scale items (see Bovaird and Koziol, 2012). Measurement models were evaluated using the fit indices CFI, RMSEA, and SRMR based on common cut-off criteria (see Kline, 2015).

The results revealed a good model fit for sensation seeking comprising the factors need for stimulation and avoidance of rest (CFI = 0.963, RMSEA = 0.044, and SRMR = 0.045), with standardized factor loadings ranging from 0.41 to 0.77. Similarly, the measurement model for cyberbullying and face-to-face bullying exhibited good model fit (CFI = 0.975, RMSEA = 0.035, and SRMR = 0.058), with standardized factor loadings ranging from 0.53 to 0.89. In sum, the results revealed a good model fit for all scales, indicating that all scales had sound measurement properties (Table 2).

**TABLE 2:** Confirmatory factor analysis (CFA) results: sensation seeking and cyberbullying and face-to-face bullying.

**Scale**	**χ^2^**	***df***	**CFI**	**RMSEA**	**SRMR**
Sensation seeking	237.49	118	0.963	0.044	0.045
Cyberbullying and face-to-face bullying	205.27	125	0.975	0.035	0.058

### Analytic Strategy

A structural equation modeling (SEM) approach based on the measurement models with ordered-categorical indicators was used to test the main hypotheses of the study (see Kline, 2015).

First, in order to investigate differential relationships between the dimensions of sensation seeking and face-to-face and cyberbullying, face-to-face and cyberbullying were predicted by need for stimulation and avoidance of rest while statistically controlling for gender, age, and social media use (Hypothesis 1). Next, to examine differences in the strength of these relationships, we tested the differences in regression slopes for need for stimulation and avoidance of rest between the face-to-face and cyber contexts for statistical significance (Hypothesis 2). To assure a common metric across face-to-face and cyberbullying, effect coding method (Little et al., 2006) was used to identify and scale the latent variables. Subsequently, to test for differences between the face-to-face and cyber contexts for each perceived context variable, we applied dependent *t*-tests with Bonferroni–Holm correction for multiple comparisons (Hypotheses 3a–3d). Lastly, to examine if perceived contextual properties affect the investigated relationships, we predicted face-to-face and cyberbullying using need for stimulation, avoidance of rest, and all four perceived context variables while also including latent interaction terms with need for stimulation and avoidance of rest for each perceived context variable and statistically controlling for gender, age, and social media use (Hypotheses 4a–4d).

Statistical analyses were conducted using Mplus version 8.1 (Muthén and Muthén, 1998–2018). Models were estimated using the robust WLSMV. We account for the hierarchical data structure (i.e., students nested within classes) by adjusting the standard errors using a sandwich estimator taking into account the non-independence of observations.

All analyses were conducted based on a statistical significance level α = 0.05.

## Results

### Descriptive Statistics

Correlation coefficients, means, and standard deviations for all variables are shown in Table 3. The results showed that cyberbullying was positively correlated with need for stimulation (*r* = 0.21), face-to-face bullying (*r* = 0.62) and social media use (*r* = 0.14). Face-to-face bullying was positively correlated with need for stimulation (*r* = 0.20), cyberbullying (*r* = 0.62), social media use (*r* = 0.10), face-to-face perceived audience size (*r* = 0.10) and face-to-face perceived lack of authorities (*r* = 0.15).

**TABLE 3:** Descriptive statistics: bivariate correlations, means, and standard deviations.

**Variable**	**1.**	**2.**	**3.**	**4.**	**5.**	**6.**	**7.**	**8.**	**9.**	**10.**	**11.**	**12.**	**13.**	**14.**	**15.**
1. Need for stimulation															
2. Avoidance of rest	–0.02														
3. Cyberbullying	**0.21**	0.06													
4. Face-to-face bullying	**0.20**	0.02	**0.62**												
5. Cyber perceived anonymity	0.06	–0.08	0.01	0.01											
6. Cyber perceived audience size	**0.12**	–0.07	–0.03	0.04	**0.42**										
7. Cyber perceived lack of authorities	**0.10**	–0.04	–0.02	–0.03	**0.31**	**0.40**									
8. Cyber perceived immediacy of reactions by others	**0.11**	0.00	0.01	0.07	**0.32**	**0.35**	**0.35**								
9. Face-to-face perceived anonymity	0.06	0.04	0.02	0.06	0.07	**0.16**	0.04	**0.14**							
10. Face-to-face perceived audience size	0.05	–0.06	0.04	**0.10**	**0.09**	–0.04	–0.01	0.02	**0.37**						
11. Face-to-face perceived lack of authorities	0.05	–0.04	0.08	**0.15**	–0.04	0.08	**0.10**	0.08	**0.38**	**0.40**					
12. Face-to-face perceived immediacy of reactions by others	0.04	–0.01	0.00	0.01	0.08	**0.11**	0.08	**0.13**	**0.59**	**0.28**	**0.40**				
13. Gender	**0.12**	**−0.13**	0.04	0.07	–0.03	–0.06	**−0.10**	–0.07	0.04	0.06	0.04	0.00			
14. Age	0.05	0.05	–0.04	–0.04	0.04	0.04	0.00	0.07	**0.13**	–0.01	0.00	0.05	**0.19**		
15. Social media use	0.06	0.02	**0.14**	**0.10**	0.06	**0.12**	0.01	0.05	0.01	0.04	0.06	0.02	**−0.16**	–0.08	

*M*	2.91	3.14	1.19	1.36	2.79	3.06	3.28	3.00	2.17	2.79	3.06	3.71	0.63	17.83	3.25
*SD*	0.74	0.82	0.38	0.49	1.21	1.29	1.26	1.23	1.34	1.30	1.32	1.31		2.13	1.39

**N* = 523; gender is coded as 0 = females and 1 = males; statistically significant results at α = 0.05 are in boldface.*

### Relationships Between Sensation Seeking and Face-to-Face and Cyberbullying

**FIGURE 1:**
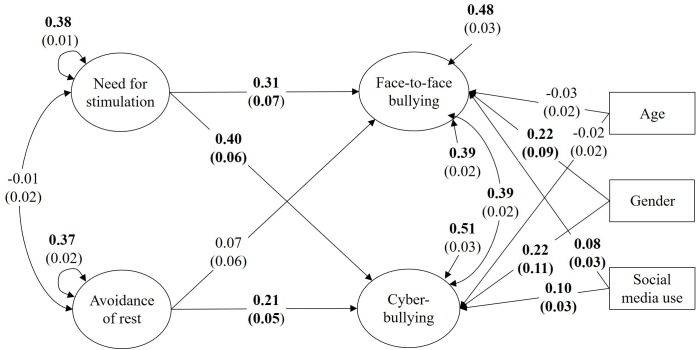
Unstandardized estimates and standard errors in parentheses. Cyberbullying and face-to-face bullying on need for stimulation and avoidance of rest with covariates age, gender, and social media use. Statistically significant results at α = 0.05 are in boldface.

The model investigating relationships between both dimensions of sensation seeking and face-to-face and cyberbullying while statistically controlling for gender, age, and social media use (Table 4) showed a good model fit [χ^2^(641) = 819.37, CFI = 0.952, RMSEA = 0.023, SRMR = 0.080]. As expected, need for stimulation was related to cyberbullying ($\hat{\beta }=0.40$, *p* < 0.001) and face-to-face bullying ($\hat{\beta }=0.31$, *p* < 0.001) when statistically controlling for gender, age, and social media use. Avoidance of rest, however, was related to cyberbullying only ($\hat{\beta }=0.21$, *p* < 0.001), but not to face-to-face bullying ($\hat{\beta }=0.07$, *p* = 0.215) (Hypothesis 1; see Figure 1).

**TABLE 4:** Structural equation modeling (SEM) results: cyberbullying and face-to-face bullying.

**Model**	**Cyberbullying**		**Face-to-face bullying**	
		
	**Est. (*SE*)**	**Std. Est.**	**Est. (*SE*)**	**Std. Est.**
**χ^2^(641) = 819.37, CFI = 0.952, RMSEA = 0.023, SRMR = 0.080**				
Need for stimulation	**0.40** (0.06)	0.32	**0.31** (0.07)	0.26
Avoidance of rest	**0.21** (0.05)	0.16	0.07 (0.06)	0.06
Gender	**0.22** (0.11)	0.28	**0.22** (0.09)	0.30
Age	−0.02 (0.02)	−0.04	−0.03 (0.02)	−0.08
Social media use	**0.10** (0.03)	0.18	**0.08** (0.03)	0.15

*Est., unstandardized estimate; SE, standard error; Std. Est., standardized estimate; gender is coded as 0 = females and 1 = males; statistically significant results at α = 0.05 are in boldface.*

The difference in regression slopes was statistically significant for need for stimulation ($\hat{\beta }=-0.10$, *p* = 0.024) and avoidance of rest ($\hat{\beta }=-0.14$, *p* = 0.002). That is, the positive relationships between both dimensions of sensation seeking and bullying were stronger in the cyber context compared to the face-to-face context (Hypothesis 2).

### Differences in Perceived Contextual Properties Between the Face-to-Face and Cyber Contexts

A series of dependent *t*-tests with Bonferroni–Holm correction for multiple testing were conducted to examine differences in perceived anonymity, perceived lack of authorities, perceived audience size, and perceived immediacy of reactions by others between the face-to-face and cyber contexts (Table 5). The results showed that perceived anonymity [*t*(521) = 8.17, *p* < 0.001, *d* = 0.36], perceived audience size [*t*(521) = 3.32, *p* = 0.002, *d* = 0.15], and perceived lack of authorities [*t*(520) = 2.94, *p* = 0.003, *d* = 0.13] were higher in the cyber context compared to the face-to-face context, while perceived immediacy of reactions by others [*t*(521) = −9.74, *p* < 0.001, *d* = 0.43] was lower in the cyber context than in the face-to-face context (Hypotheses 3a–3d).

**TABLE 5:** Dependent *t*-tests results: cyber and face-to-face contexts.

**Variable**	**Cyber context**		**Face-to-face context**		**Dependent *t*-test**	**Cohen’s *d***
				
	***M***	***SD***	***M***	***SD***		
Perceived anonymity	2.79	1.21	2.17	1.34	*t*(521) = 8.17, *p* < 0.001	0.36
Perceived audience size	3.06	1.29	2.79	1.30	*t*(521) = 3.32, *p* = 0.002	0.15
Perceived lack of authorities	3.28	1.26	3.06	1.32	*t*(520) = 2.94, *p* = 0.003	0.13
Perceived immediacy of reactions by others	3.00	1.23	3.71	1.31	*t*(521) = −9.74, *p* < 0.001	0.43

*Bonferroni–Holm correction for multiple testing was applied.*

### Interactions Between Perceived Contextual Properties and Sensation Seeking

The model including latent interaction terms between all four context variables and need for stimulation and avoidance of rest was estimated while statistically controlling for gender, age, and social media use (Table 6).

**TABLE 6:** SEM with latent variable interaction results: cyberbullying and face-to-face bullying.

**Model**	**Cyberbullying**		**Face-to-face bullying**	
		
	**Est. (*SE*)**	**Std. Est.**	**Est. (*SE*)**	**Std. Est.**
Need for stimulation	**0.42** (0.21)	0.27	**0.26** (0.11)	0.19
Avoidance of rest	**0.25** (0.12)	0.16	0.11 (0.08)	0.08
Perceived anonymity	**−0.52** (0.22)	−0.17	0.01 (0.17)	−0.00
Perceived audience size	−0.10 (0.09)	−0.06	**0.25** (0.09)	0.17
Perceived lack of authorities	0.16 (0.09)	0.05	−0.27 (0.14)	−0.11
Perceived immediacy of reactions by others	0.10 (0.11)	0.05	−0.04 (0.08)	−0.03
Need for stimulation × perceived anonymity	−0.01 (0.17)	0.04	−0.16 (0.12)	−0.09
Avoidance of rest × perceived anonymity	−0.10 (0.13)	−0.06	0.08 (0.12)	0.05
Need for stimulation × perceived audience size	−0.03 (0.09)	−0.03	0.03 (0.06)	0.03
Avoidance of rest × perceived audience size	0.02 (0.07)	0.02	−0.11 (0.07)	−0.11
Need for stimulation × perceived lack of authorities	−0.20 (0.14)	−0.11	−0.05 (0.13)	−0.03
Avoidance of rest × perceived lack of authorities	0.11 (0.13)	0.06	**0.21** (0.10)	0.12
Need for stimulation × perceived immediacy of reactions by others	−0.09 (0.07)	−0.07	0.03 (0.07)	0.03
Avoidance of rest × perceived immediacy of reactions by others	0.00 (0.08)	0.00	−0.05 (0.11)	−0.05
Gender	0.47 (0.33)	0.21	0.41 (0.25)	0.20
Age	−0.11 (0.09)	−0.11	−0.07 (0.04)	−0.07
Social media use	**0.31** (0.16)	0.19	0.14 (0.08)	0.09

*Model fit is not available in latent interaction models. Est., unstandardized estimate; SE, standard error; Std. Est., standardized estimate; gender is coded as 0 = females and 1 = males; statistically significant results at α = 0.05 are in boldface.*

With respect to cyberbullying, no statistically significant interactions between either dimension of sensation seeking and any of the four perceived context variables were found. For face-to-face bullying, a statistically significant interaction between avoidance of rest and perceived lack of authorities was found ($\hat{\beta }=0.21$, *p* = 0.020), indicating that the (non-significant) relationship between avoidance of rest and face-to-face bullying increases with an increasing perceived lack of authorities. All other interaction effects were statistically insignificant (Hypotheses 4a–4d).

## Discussion

Sensation seeking is widely considered a risk factor for aggressive behavior (Zuckerman, 2007; Wilson and Scarpa, 2011; Bacon et al., 2014). However, studies investigating the relationship between sensation seeking and bullying – a subtype of aggressive behavior (e.g., Smith, 2004) are scarce and have mostly operationalized sensation seeking in ways that also partially measure antisocial behavior, creating a risk of obtaining tautological findings. Moreover, as it remains questionable whether bullying in the face-to-face context and in cyberspace may be considered equivalent (Olweus, 2012), the context (face-to-face, cyberspace) should not be neglected when investigating bullying (e.g., Graf et al., 2019).

Thus, the present study examined sensation seeking’s associations with both face-to-face and cyberbullying. Moreover, our operationalization of sensation seeking as a need (Cattell, 1979), without measuring concrete antisocial behaviors, allowed us to ensure that this study’s findings are not biased due to tautologies. Hence, we avoided a situation in which we regressed self-descriptions of concrete antisocial behavior – as is partially the case in the most common measurement instruments for sensation seeking (e.g., SSS-V; Zuckerman et al., 1978) – on self-descriptions of antisocial behavior used to operationalize face-to-face and cyberbullying behavior. Consequently, this approach improved the interpretability of our results.

Moreover, by investigating differential relationships between sensation seeking and face-to-face and cyberbullying, this study contributes to recent discussions about similarities and differences between face-to-face and cyberbullying. Additionally, we provide deeper insights into how contextual properties may account for sensation seeking’s differential role in face-to-face and cyberbullying by examining perceived differences in contextual properties between contexts (face-to-face vs. cyberspace) and how the perception of these contextual properties may shape the relationships between sensation seeking and bullying in both contexts.

Overall, our results indicate that sensation seeking should be recognized as a risk factor for both face-to-face and cyberbullying. Moreover, we found that sensation seeking plays a stronger role in cyberbullying than in face-to-face bullying. Additionally, we observed differences between face-to-face communication and CMC in students’ perceptions of all investigated contextual properties. However, perceived contextual properties had no influence on the significant relationships between sensation seeking and face-to-face and cyberbullying.

Although we hypothesized positive relationships between both dimensions of sensation seeking and face-to-face and cyberbullying (Hypothesis 1), we found that avoidance of rest solely predicted cyberbullying. Furthermore, as hypothesized (Hypothesis 2), we observed a significantly stronger association between both dimensions of sensation seeking and cyberbullying compared to face-to-face bullying. These results indicate that sensation seeking might be differentially relevant as a risk factor for face-to-face vs. cyberbullying, playing a more important role for cyberbullying. The unique relationship between avoidance of rest and cyberbullying might be explained by findings suggesting a relationship between boredom, defined as “a state of relatively low arousal and dissatisfaction which is attributed to an inadequately stimulating environment” (Mikulas and Vodanovich, 1993, p. 1), and aggressive behavior (e.g., Rupp and Vodanovich, 1997). Obviously, cyberbullying is just one possible behavior that can counteract the “restless, irritable feeling” (Barbalet, 1999, p. 631) resulting from a situation which holds no appeal (Barbalet, 1999). However, following the assumption that communication in cyberspace may take place in less stimulating environments compared to face-to-face communication, e.g., due to a smaller set of possible actions or greater remove from the social situation (see social presence theory; Gunawardena, 2004), future research should investigate state experiences such as boredom in relation to bullying incidents. We propose that state boredom could be a mediator variable between avoidance of rest and cyberbullying. In contrast, this might not be the case for face-to-face bullying or only be so to a lesser extent.

Moreover, our findings show that theoretically assumed differences between contexts are indeed perceived as such (Hypotheses 3a–3d). Anonymity, audience size, and lack of authorities were perceived as higher in CMC compared to face-to-face communication. In contrast, the immediacy of reactions by others was perceived as higher in face-to-face communication than in communication with electronic devices. We found the strongest differences between the cyber and face-to-face contexts for perceived anonymity and perceived immediacy of reactions by others, with small-to-medium effect sizes. Further research on contextual differences between face-to-face and cyberbullying may wish to address these specific contextual properties in greater detail. For example, perceived anonymity and perceived immediacy of reactions by others with respect to the target of the bullying behavior, the authorities, or even bystanders might be considered (regarding anonymity, see also Wright, 2013).

While we found differential relationships between the two dimensions of sensation seeking and both forms of bullying, as well as differences in perceived contextual properties, we only found one moderation of perceived contextual properties on the investigated relationships (Hypotheses 4a–4d). A perceived lack of authorities reinforced the relationship between avoidance of rest and face-to-face bullying, as hypothesized, but did not play the same role for cyberbullying. Moreover, the practical relevance of this finding is questionable due to the non-significant relationship between avoidance of rest and face-to-face bullying. Nevertheless, while interpretations should be tentative, this result may indicate the importance of being aware of authorities when it comes to preventing face-to-face bullying incidents motivated by avoidance of rest. In contrast, the protective role of the perceived presence of authorities might not be that important in cyberspace. In combination with our finding that authorities are perceived as more present in face-to-face context than in cyberspace, this might partially explain the non-significant relationship between avoidance of rest and face-to-face bullying compared to the significant positive association with cyberbullying. Further, the lack of significant interactions between need for stimulation and the perceived contextual properties investigated in our study indicates that need for stimulation might be a common dispositional risk factor for face-to-face and cyberbullying resistant to situational influences.

The fact that we only found one significant interaction (reinforcing a non-significant relationship), in contrast to our hypotheses that all perceived contextual properties would affect the relationships investigated in this study, indicates that further studies should not solely consider single contextual properties but should take a more holistic approach.

With respect to the covariates, we found no relationships between age and self-reported face-to-face and cyberbullying after controlling for both dimensions of sensation seeking and all other covariates. Although the existing literature suggests variations in the prevalence rates of face-to-face and cyberbullying across different age groups (e.g., Kowalski et al., 2014), we were unable to find such associations. One explanation for this finding could be the fact that we controlled for a set of other variables in the model. Another explanation could be the unequal age distribution in our sample (i.e., almost two-thirds of the participants were between 16–18 years old). However, our results were in line with recent findings concerning gender and social media use. Like Best et al. (2014), we found statistically significant associations between social media use and both face-to-face and cyberbullying (even while controlling for both dimensions of sensation seeking and all other covariates). Moreover, we found greater involvement in both face-to-face and cyberbullying among boys, as previously suggested (e.g., Kowalski et al., 2014).

### Limitations

First, we acquired our information via self-reports. While some studies criticize the susceptibility of self-reports to social desirability mechanisms (Beran et al., 2012), the literature has repeatedly shown self-reports to be a valid method to assess bullying and personality (Pellegrini and Bartini, 2000; Lee and Ashton, 2004). Nevertheless, taking a multi-informant approach in further studies would enrich the depth of information available. In addition, we draw our conclusions based on cross-sectional data, meaning that we are not able to make causal interpretations. Therefore, future studies should replicate our findings using longitudinal data or experimental designs. Moreover, we used a variable-oriented approach. The results and conclusions drawn from our study could be complemented and extended with person-oriented approaches such as latent-class analyses or cluster analyses (see von Eye and Spiel, 2010). Finally, we operationalized the perceived contextual properties using one-item measures. Thus, the reliability and validity of these measures could be subject to critique. Follow-up research should develop measures to assess contextual properties in more detail.

## Conclusion

Our study provides useful insights into the perceived differences in contextual properties between face-to-face and online communication, providing a base for future studies. Moreover, our study adds to the current literature discussing similarities and differences between face-to-face and cyberbullying by demonstrating differential relationships between dimensions of sensation seeking and face-to-face and cyberbullying, with sensation seeking playing a more negative role in cyberbullying. By demonstrating differences in the associations between an intrapersonal risk factor and face-to-face and cyberbullying, our findings provide additional evidence to inform the evidence-based development of effective prevention and intervention strategies that consider the contexts (face-to-face vs. cyberspace) in which bullying appears.

## Data Availability

The datasets generated for this study are available on request to the corresponding author.

## Ethics Statement

This study was carried out in compliance with ethical standards of the Austrian Federal Ministry of Health (1995) and the American Psychological Association (2010). Prior to participation, students gave written informed consent. The consent form informed participants about duration, procedure, and goals of this study. Participants were guaranteed anonymity and confidentiality of their data and were informed that participation was voluntary and could be withdrawn at any point of the questionnaire. According to Austrian and European (EU) law, approval of an ethics committee was not necessary as this study did not involve patients, was non-invasive, and participation was voluntary and anonymous.

## Author Contributions

DG and CS developed the study concept. DG and TY prepared the draft manuscript and CS provided critical revisions. All authors contributed meaningfully to the manuscript and the study design, analyzed or interpreted the data, and approved the final version of the manuscript for submission.

## Conflict of Interest Statement

The authors declare that the research was conducted in the absence of any commercial or financial relationships that could be construed as a potential conflict of interest.
